# Bio-Based Alternatives to Phenol and Formaldehyde for the Production of Resins

**DOI:** 10.3390/polym12102237

**Published:** 2020-09-28

**Authors:** P. R. Sarika, Paul Nancarrow, Abdulrahman Khansaheb, Taleb Ibrahim

**Affiliations:** 1 Department of Chemical Engineering, American University of Sharjah, PO Box 26666, Sharjah, UAE; sreghunadh@aus.edu (P.R.S.); italeb@aus.edu (T.I.); 2 Khansaheb Industries, Airport Road, Rashidiya, PO Box 13, Dubai, UAE; abdulrahman@khansaheb.ae

**Keywords:** phenol-formaldehyde resin, resol resin, lignin, tannin, cardanol, hydroxymethylfurfural, furfural, glyoxal, sustainable resin

## Abstract

Phenol–formaldehyde (PF) resin continues to dominate the resin industry more than 100 years after its first synthesis. Its versatile properties such as thermal stability, chemical resistance, fire resistance, and dimensional stability make it a suitable material for a wide range of applications. PF resins have been used in the wood industry as adhesives, in paints and coatings, and in the aerospace, construction, and building industries as composites and foams. Currently, petroleum is the key source of raw materials used in manufacturing PF resin. However, increasing environmental pollution and fossil fuel depletion have driven industries to seek sustainable alternatives to petroleum based raw materials. Over the past decade, researchers have replaced phenol and formaldehyde with sustainable materials such as lignin, tannin, cardanol, hydroxymethylfurfural, and glyoxal to produce bio-based PF resin. Several synthesis modifications are currently under investigation towards improving the properties of bio-based phenolic resin. This review discusses recent developments in the synthesis of PF resins, particularly those created from sustainable raw material substitutes, and modifications applied to the synthetic route in order to improve the mechanical properties.

## 1. Introduction

Phenolic resin synthesized from phenol and formaldehyde continues to adorn the resin industry, over a century after its first development, due to its versatile properties and performance in a wide variety of applications. The production and commercial applications of phenolic resin quickly spread all over the world after its first commercial production in Germany in 1909. Excellent mechanical properties, flame retardancy, flexibility, low cost, high thermal stability, and water and chemical resistance have made it the material of choice for applications in the aerospace industry, as adhesives in particleboard manufacturing units [1], as paints and coatings, in the manufacture of insulating foams and in the electrical and lighting industry [2,3]. Throughout the 20th century, phenolic resins have extended into several sectors owing to the wide range of favorable properties, which can be tailored according to the end use. In order to make them suitable for a wide range of applications, many synthetic modifications have been adopted. For example, the mechanical properties have been improved by incorporating various fibers and fillers [4,5], and thermal properties have been enhanced by using substituted phenols [6,7,8]. New modifications are continually being developed, allowing the creation of new valuable products made from phenolic resin.

Despite the widespread popularity and breadth of application of PF resins, in recent decades, there has been a significant drive towards partially or fully replacing the raw materials, phenol and formaldehyde, in the synthetic process. There are three main motivations for such a replacement: (1) moving towards more sustainable bio-sources of raw materials; (2) improving health and safety during manufacture and end use; and (3) enhancing the performance characteristics of the resin. These motivations are discussed in the following paragraphs.

Petroleum is currently the main source of raw materials for phenol and formaldehyde commercial production. Phenol, the main raw material in phenolic resin, was first manufactured commercially by distillation of coal tar. The cumene route, which involves the synthesis of phenol from benzene and propylene, via the intermediate cumene, replaced the distillation process as the most common industrial process for phenol production, due to its favorable economics. Several other synthetic routes have been developed over the years for the phenol production from petroleum derived raw materials [9]. The other raw material in the phenolic resin manufacturing, formaldehyde, is also produced mainly from petroleum resources [9]. However, petroleum is a non-renewable natural resource and its continued use as a source of bulk chemicals for manufacturing is not sustainable in the long term. Therefore, many researchers are shifting their focus towards biomass as a renewable source of carbon-based reagents, and its extensive and accurate use will solve the issues associated with petroleum-based resources. Advancement in chemical technology offers a sustainable manufacturing paradigm by providing easy conversion of biomass into valuable chemicals and biofuels [10]. In the future, more chemicals will be produced from renewable biomass to deal with the depletion of natural resources.

Biorefineries play an important role in converting biomass into value-added products, and lignocellulose serves as the main primary resource for further synthesis of bioderived materials. Phenolic compounds, alcohols, and furan-based compounds are some of the chemicals produced from lignocellulose through the biorefineries [11,12,13]. The main advantage of lignocellulose is its abundance, renewability, and its free availability as a waste product from the food industry, for example [14]. A number of reviews have been published in recent years describing the methods used to convent lignocellulose into value-added products, chemical, and physical pretreatment methods [14,15,16].

One of the potential ecofriendly substitutes for phenol is lignin, the main component in lignocellulosic biomass [17,18,19]. Lignin has a phenolic structure, and it is now widely used in the production of phenolic resin. Cardanol [8,20], tannin [21,22], gallic acid [23], and bio-oils [24,25] are also used as substitutes for phenol in resin production. Formaldehyde can also be substituted for alternative raw materials from bioresources. The major challenge in replacing formaldehyde is finding an alternative with a high reactivity and low molecular weight. Hydroxymethylfurfural (HMF), furfural [26], terephthalaldehyde [27], and glyoxal [28] have all been reported as potential replacements for formaldehyde in the production of phenolic resin.

The second motivation for replacing phenol and formaldehyde in the manufacture of PF resin is related to health and safety. While PF resins and their associated products are considered relatively safe and stable materials, the raw materials used in their manufacture have significant safety issues. The World Health Organization (WHO) has listed formaldehyde as carcinogenic [29], and the US Occupational Safety and Health Administration (OSHA) has strictly limited their permissible exposure levels due to the health risk of these chemicals. According to the European Chemical Agency (ECHA), both phenol and formaldehyde are mutagenic, carcinogenic, and reprotoxic [30]. This creates significant challenges during the manufacturing process for PF resins, and expensive safety measures are necessary to prevent worker exposure. Furthermore, residual formaldehyde can remain in the PF resin products, leading to the potential for low level, long term exposure in homes and offices, where PF products are commonplace. Therefore, in order to improve the safety of PF resin manufacture and end use, many researchers are investigating alternative, less harmful, raw materials such as lignin and HMF, for example.

The third motivation for modifying the PF synthesis is to enhance performance characteristics of the end products. While PF resin already has highly favorable properties, there is always a drive to continue improving upon these to develop resins with improved mechanical properties, heat resistance, thermal insulation, and reduced formaldehyde emissions. Such improvements have the potential to extend the range of applications of PF resins, and enhance their performance in existing applications. However, switching to more sustainable raw materials while maintaining the desirable performance characteristics and reactivity of phenol and formaldehyde is a major challenge. Although much of the work on replacing phenol or formaldehyde has led to an improved environmental profile at the expense of some performance, some recent research has shown that partially replacing phenol and formaldehyde in PF resin manufacture with biomass-derived materials can potentially lead to improvements in properties such as mechanical strength while also reducing the problems associated with PF resin manufacture.

This review focuses on the recent developments in the synthesis and applications of phenolic resin. In particular, it will discuss the classification of phenolic resin based on the synthesis conditions, biobased substitutes for phenol, and formaldehyde, and the modifications performed on the reagents to enable the production of resin from more sustainable resources for a variety of industrial applications.

## 2. Materials and Methods

**Figure 1:**
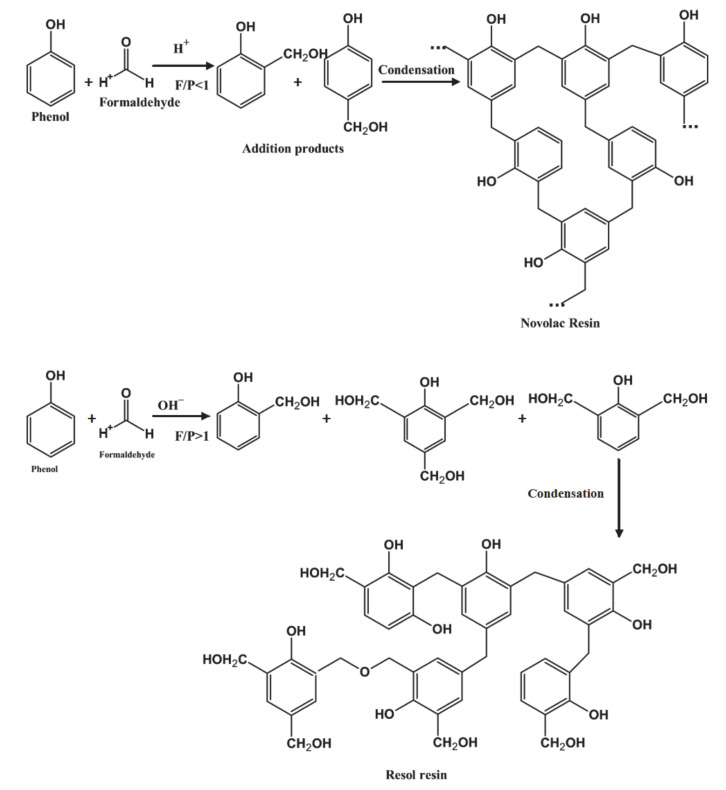
Schematic representation of resol and novolac resin synthesis.

Phenol-formaldehyde resin is synthesized from phenol and formaldehyde by acidic or basic catalytic reactions. The structure and properties of the phenol–formaldehyde resin are highly dependent on the reaction conditions, the type of catalysts, and the molar ratio of reactants. Phenolic resin is classified into novolac or resol resin based on the pH and phenol to formaldehyde ratio used in the reaction. Resol type resins are formed under basic conditions when the molar amount of formaldehyde exceeds that of phenol. They are thermosetting resins with methylol and hydroxyl group capable of forming a cross-linked network without the use of additional curing agents. This, in itself, represents a significant environmental benefit over novolac resins, since most curing agents used have toxic effects. If the resin is prepared under acidic conditions, and with an excess of phenol compared to formaldehyde, novolac type resin will be formed. The shelf life and stability of novolac resins are higher compared with those for resol resin. However, an additional catalyst is required to cure novolac resins [31]. The reaction mechanisms of both types of phenolic resin were described in a recently published review [1]. Schematic representations of resol and novolac resin synthesis are shown in Figure 1.

## 3. Phenol Substitutes for PF Resin

The largest consumption of phenol–formaldehyde resin happens in the wood product industry, where it is used as an adhesive in the production of particleboard, plywood, fiberboard, MDF board, and other products. Production of phenol–formaldehyde resin can be volatile due to the fluctuating price of petroleum-derived phenol raw material. Hence, there are economic as well as environmental incentives to develop a renewable phenol resource. Biomass is the best alternative for phenol production because it is renewable, often readily available as a waste material, and yields products with lower carbon footprint. Extraction of monomers and chemicals from the natural resource is sometimes challenging due to variability in the chemical content and composition of the biomass, high extraction and processing costs, low extraction yields, and the requirement of non-destructive extraction techniques. The main natural resources of phenols are lignin [32], tannin [33], cardanol [34], palm oil, and coconut shell tar. Many phenolic resins have been developed based on these natural substitutes.

### 3.1. Lignin-Based PF Resins

**Figure 2:**
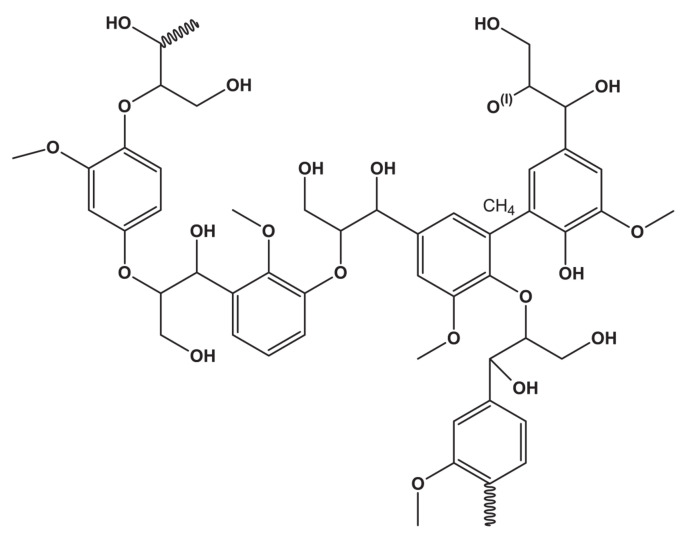
Structure of lignin.

Lignin obtained from the lignocellulose biomass is an attractive substitute for fossil based raw materials. Lignocellulose is made up of three polymers: lignin, cellulose, and hemicellulose. Their relative compositions vary in different biomass resources. Lignin consists of three phenyl-propanols i.e., p-coumaryl alcohol (p-hydroxyl-phenyl propanol), coniferyl-alcohol (guaiacyl-propanol), and sinapyl-alcohol (syringyl-propanol) [35]. Pyrolysis [36] or direct liquefaction processing [37] have been utilized to separate the phenyl propanol units in the lignin. The chemical structure of lignin is shown in Figure 2.

A number of processes have been developed to separate the components of lignocellulose. These include: extraction of lignin at high temperature and pressure; depolymerization of the lignocellulose structure using mineral acids at elevated temperatures, the dissolution of cellulose, followed by reprecipitation, steam explosion/ethanol process [38], prehydrolysis and extraction process [39], SPORL process [40], ethanol organosolv and ultrafiltration process [41], enzymatic [42], and catalytic conversion [43].

Sulphur containing and sulfur free lignin are produced from lignocellulose materials. Kraft lignin and lignosulfonates are sulfur-containing lignins, which are obtained mainly from the pulping and paper making industries. The mechanical properties of Kraft and lignosulfonate modified PF resin is inferior to that of PF resin because of the low reactivity of these types of lignin with formaldehyde in comparison to phenol. In addition, the processing leads to the corrosion of the equipment, and sulfur causes environmental issues [44]. Soda-anthraquinone, organosolv, a steam-explosion, oxygen delignification, and hydrolysis processing can be used to produce sulfur-free lignin [45].

Lignin-based phenolic resins have been reported in the literature. The large number of hydroxyl groups on the structure of lignin makes it a suitable candidate to replace phenol during phenolic resin production [46]. However, the steric hindrance and the abundant methoxy content in lignin reduce the practical use [47]. Lignin can be used as a raw material for the production of PF resin in three forms: raw lignin, purified lignin, and chemically modified lignin. The raw lignin is the material obtained directly from biorefineries. Due to the low reactivity of raw lignin with formaldehyde in PF resin synthesis, the substitution rate is low. Hence, purified lignin is typically adopted for the synthesis of lignin modified PF resin. In the third approach, the reactivity of lignin is further improved by chemical modification. The abundance of phenolic and aliphatic hydroxyl groups on lignin facilitates chemical modification. Methylolation [48], phenolation [49], and demethylation [50] are the three main methods used to improve the reactivity of lignin [51]. In methylolation, hydroxymethyl groups are introduced to the lignin molecule. El Mansouri et al. modified rice straw lignin by hydroxymethylation to increase the number of reactive sites. The higher reactivity of this modified lignin enabled the substitution of 20–50% of phenol in phenol–formaldehyde resin synthesis [52]. Wang et al. substituted 50% of phenol in phenol–formaldehyde adhesive using methylolated lignin liquor obtained from steam treated corn stalk. The demethylation of lignin has been performed with hydrogen iodide [53], hydrogen chloride [54], sulphur dioxide [55], and sodium sulphite [56]. Demethylation with sodium sulphite was found to enhance the efficiency and reactivity of lignin. The PF resin prepared with demethylated resin shows a faster curing rate, shorter gel time, higher reactivity, and lower formaldehyde emissions [56]. Podschun et al. reported the phenolation of beech organosolv lignin to increase the number of potential cross-linking sites. The degree of phenolation was found to depend significantly on the phenol/lignin feed ratio, catalyst content, reaction time, temperature, and water and solvent content [57]. 

Depolymerization is another approach to convert complex lignin molecule into a small molecule or oligomers to expand its application. Depolymerization of lignin can be base-catalyzed, acid-catalyzed, metallic catalyzed, ionic liquids-assisted, or supercritical fluids-assisted. Base catalyzed lignin depolymerizations are usually carried out at high temperatures and pressures [21,58,59]. Acid catalyzed lignin depolymerization also requires harsh reaction conditions, which increases the reaction cost [60]. Metal catalyzed lignin depolymerizations are highly selective and cost effective. Among various metal catalysts, Ni- and Pt- based catalysts are commonly used for lignin depolymerization [61,62,63]. Even though ionic liquids [64,65] and supercritical fluids-assisted depolymerizations [66] provide high selectivity, their high cost hinders widespread application.

Unlike other biopolymers, lignin exhibits an irregular structure, which depends on the vegetal species of origin. The properties of lignin substituted resins strongly depend on the source and reactivity of the lignin. Several types of lignin have been investigated for use in PF resins, such as Kraft, soft wood, wheat straw, and corn stover lignins. 

Abdelwahab et al. incorporated Kraft lignin into PF resin and studied the effect of percentage of substitution on the adhesive properties of the resin. The adhesive strength was found to increase with Kraft lignin concentration, reaching the maximum strength at 90% substitution. The bagasse raw material and the method used in the pulping process are responsible for the relatively high reactivity of Kraft lignin [67] with formaldehyde. Lignin obtained from wheat straw by the organosolv pretreatment method with 50–70% phenol substitution yielded similar physicochemical properties to standard PF resin [68].

Soft wood lignins offer significant advantages over hard woods and crop residues in the production of lignin-PF resin due to the higher content of highly reactive guaiacol units. Wang et al. used lignin obtained from white pine dust via the organosolv extraction method to substitute 25–70% of the phenol in PF resin production. Larger substitution ratio resulted in a delayed curing process, due to the lower reactivity of the lignin, whereas thermal cure at a lower temperature was observed when the substitution was less than 50% compared to the phenol–formaldehyde resin. At a lower substitution rate, the curing was found to be governed by phenol reactivity and a small amount of lignin with a large number of phenylpropane units was also favorable to the thermal cure [69]. Kalami et al. replaced 100% of the phenol with lignin for the synthesis of resole resins. The resin was used as adhesive for making plywood, and the shear strength of the resulting samples was found to be similar to that made from conventional PF resin-based plywood. The lignin was obtained via the cellulosic bioethanol process through dilute-acid pretreatment, and enzymatic hydrolysis from corn stover [70].

The residue produced in the bioethanol industry is mainly composed of lignin. Lignin obtained from the different biorefinery processes has a different chemical structure and reactivity. Wei Zhang et al. used four kinds of biorefinery residues to modify phenol–formaldehyde resin and the bioethanol biorefinery residue (ER) showed higher reactivity with formaldehyde, due to the presence of a high number of hydroxyl groups. Of phenol in PF resin 50% was substituted with ER and the ERPF resin shows a low formaldehyde emission of 0.32% and bonding strength of 98 MPa, which is relatively high in comparison with other biorefinery substituted resins [71]. Impurities such as ash and carbohydrates are also present in this lignin; these need to be removed to improve its reactivity. Yanqiao Jin et al. utilized enzymatic hydrolysis lignin (EHL) to produce modified phenol–formaldehyde resin. Phenol was partially substituted with EHL during the PF resin production and the properties of plywood glued with the EHL modified PF resin adhesive was found to meet the Chinese National Standard GB/T14732-2006 when the replacement percentage of phenol by EHL was in the range of 5–20 wt % [72]. Qiao et al. adopted another technique to improve the reactivity of bioethanol production lignin residue; initially the residue was pretreated to isolate lignin and phenolation was performed in the subsequent step. The modified lignin was used to substitute phenol in the preparation of PF resin and the properties of the resin were compared with enzymatic hydrolysis lignin-modified phenol–formaldehyde resin (EHL-PF) [73]. It was found that the purification process enhanced the phenol substitution rate without compromising the adhesive strength of the resin.

Domínguez et al. studied the properties of phenol–formaldehyde resin affected by lignin substitution. Various properties such as structure, reactivity, thermal stability, and rheological properties of commercial resol resin and a bioresin formulated partially with modified ammonium lignosulfonate were compared. It was found that lignin modified resin displayed better mechanical properties and higher thermal stability than that of standard PF resin [74]. However, even though lignin is an attractive substitute for phenol in the production of phenol–formaldehyde resin, its lower reactivity and the additional energy and cost required for its modification hinder its use in industrial applications. Liu et al. found a new method based on an oxalic acid/choline low transition temperature mixture (LTTM) at low temperature to activate chemical groups in lignin. The modification method increased the phenolic –OH content from 1.75 to 3.01 mmol/g and decreased the –OCH3 content from 10.86 to 8.57 wt %. The modified lignin was partially substituted with phenol and reacted with furfural to prepare lignin-furfural (PFU) resins. The lignin-PFU resin with 50% lignin substitution showed a high bond strength (up to 1.84 MPa), low free phenol content, higher curing rate and higher thermal stability than phenol-furfural resin [75]. The various lignin substituted resins and their associated properties are summarized in Table 1.

### 3.2. Tannin-Based PF Resins

**Figure 3:**
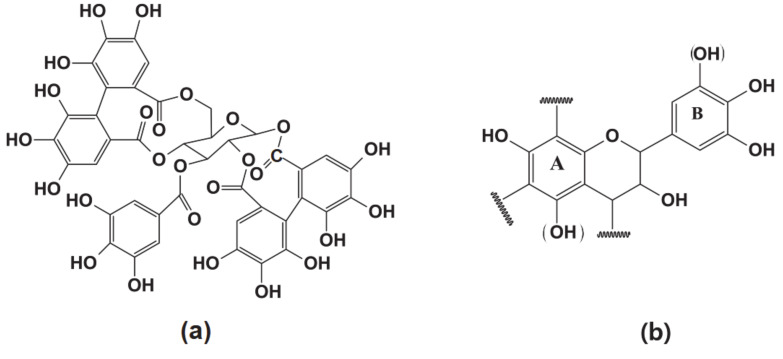
Structures of (**a**) hydrolysable tannins and (**b**) condensed (flavonoid) tannins.

Another important green substitute for phenol in PF resin synthesis is tannin, a polyphenolic biomolecule present in the bark and wood of trees. It is extracted from the bark at different temperatures using hot water and is extensively used in the manufacturing of heavy leathers. Tannins are classified into two categories: hydrolysable and condensable tannins, based on their chemical properties. The structures of hydrolysable and condensable tannins are shown in Figure 3. Hydrolyzable tannins contain a mixture of phenols, which can be hydrolyzed into phenolic acid, and carbohydrates, by treatment with weak acids and bases [79]. Chestnut (*Castanea sativa*), myrabolans (*Terminalia* and *Phyllantus* tree species), divi–divi (*Caesalpina coraria*), tara, algarobilla, valonea, and oak trees contain hydrolysable tannins [80]. They are divided into gallotannins and ellagitannins, which release gallic acid and ellagic acid, respectively, upon hydrolysis. Hydrolysable tannins have a more complex structure and lower reactivity than condensable tannins. Despite their lower reactivity, hydrolysable tannins are used for substituting phenol in PF resin. Valonea (*Quecus acutissima* Carr) tannin (VT) has been successfully used to partially replace phenol in phenol-tannin-formaldehyde (PTF) resin synthesis, and the resulting resin displayed a reduced curing time and lower formaldehyde emission [81].

Almost 90% of the commercial tannins are obtained from condensable tannins, which contain two aromatic rings with different hydroxyl groups. The reactivity of the tannin depends on the positions of the hydroxyl group. The efficiency of tannin extraction from plants depends on the plant species and the solvent used for extraction. It can also be obtained from agricultural waste [82], grapes skin [83], and fruit residues [84]. Tannins extracted from larch [85], pine [86], bark of wattle [87], and quebracho [88] have been used to produce resin with similar properties to PF resin. Li et al. studied the reactivity of condensable and hydrolysable tannin towards formaldehyde in PF resin synthesis. Resins substituted with larch (condensable) and valonia (hydrolysable) tannins were prepared and the properties such as viscosity, solid content, gel time, and free formaldehyde content were compared. All the properties of larch tannin-based resin were found to be better and the formaldehyde emissions lower [89]. Due to their higher reactivity, condensable tannins are more commonly used in resin production than hydrolysable tannins.

García et al. developed novolac resin from maritime pine (*Pinus pinaster* Ait.) bark condensed tannin with a number of aldehydes in different molar ratio and investigated the influence of the aldehyde structure on the resin properties. It was found that increasing the chain length affected the glass transition temperature and density of the resin. Thermal decomposition temperature of the resin increased up to 80 °C with an increase in aldehyde chain length [86]. Li Cheng et al. developed phenol–tannin–urea–formaldehyde (PTUF) resins by copolymerization of tannin, urea, phenol, and formaldehyde. Due to the steric hindrance in tannin, the co-polycondensation between the hydroxyl becomes difficult and introduction of urea helps to connect the phenolic sections with tannin molecules. The properties of the plywood bonded with optimized tannin substituted resin met the Chinese National Standard (GB/T9846.1-9846.8-2004) for E0 class plywood [90].

Jahanshaei et al. substituted 10–30% of phenol with tannin extracted from the bark of the oak tree in phenol formaldehyde resin. Tannin substitution at 30% was found to considerably reduce formaldehyde emission from particleboard, with around 20% reduction in the modulus of rupture (MOR) and 10% reduction in the modulus of elasticity (MOE). Even though the tannin resin with up to 30% substitution shows significant reduction in the internal bond (IB) strength, water absorption, and thickness swelling, the values are still higher than the European standard (EN) for exterior particleboard [91]. Lagel et al. used hydrolyzable chestnut tannin to replace 30% of phenol in PF resin, and the resultant resin exhibited increased compressive strength [92]. Abdalla et al. substituted 50% of phenol by tannin from acorns of valonia oak in PF resin synthesis and the resin was used as an adhesive in particle board manufacturing [93]. Higher tannin substitution also increases the viscosity of the resin. Hafiz et al. studied the effects of tannin substitution on the curing behavior and the thermal properties of PF resin. The viscosity of the resin was found to increase with an increase in tannin substitution, and the PF with the highest tannin substitution, 40%, showed the lowest gel time. The PF resin with 30% tannin substitution exhibited the lowest curing temperature, high thermal stability, strongest maximum rigidity as well as high shear bond strength as compared to the other tannin substituted resins [94].

Several depolymerization techniques have been utilized to convert tannin to oligomers and monomers in order to decrease steric hindrance and enhance chemical reactivity. Zhang et al. depolymerized larch tannin into condensed tannin by sulfonation pretreatment, and used it for PF resin preparation [95]. Recently, Li et al. depolymerized acacia mangium tannin under acidic conditions in the presence of the nucleophilic reagent, 2-methylfuran. The prepared depolymerized tannin-substituted PF resin (DTPF) was found to have a fast curing rate, good adhesion properties, low formaldehyde emission, and high thermal stability. Compared with tannin-phenol-formaldehyde (TPF) resin, DTPF resin shows a 67.6% decrease in gel time, while the mass loss after hydrolysis decreased by 26.3%, the bonding strength increased by 63.6%, and the formaldehyde emission decreased by 64.4% [21].

Tannin substituted phenolic resins have been used as adhesives in the wood industry and for making insulation foams. The polyhydroxyphenyl groups in tannin have a great affinity towards metal ions, and this property enables them to be used as ion adsorbents. Mulani et al. reported the ability of tannin-aniline-formaldehyde resin for arsenic removal from contaminated water. The complexation between arsenic ion and phenolic –OH groups on the resin enables the purification of arsenic contaminated water [96]. Tannin-phenol-formaldehyde resins produced using tannin from the dried fruit of *Terminalia chebula* (Aralu) were shown to adsorb bivalent cations such as Zn^2^⁺, Pb^2^⁺, Ca^2^⁺, Mg^2^⁺, and Cu^2^⁺ [97]. Zhang and coworkers, partially replaced formaldehyde with furfural in tannin-based phenolic resin with the goal of reducing the formaldehyde emission from the resin. In this research, a new tannin modification method was established to increase the efficiency between the tannin and furfural. Both tannin and furfural were pretreated, to depolymerize the tannin and open the furan ring of the furfural respectively, with the aim of increasing the reactivity between them. Pretreatment increased the substitution ratio with a decrease in free formaldehyde content from 5.57% to 1.42%. Incorporation of furfural into the resins was found to increase the thermal stability of the resin [98]. The tannin-substituted resin and their performance characteristics are summarized in Table 2.

### 3.3. Cardanol-Based Phenolic Resin

**Figure 4:**
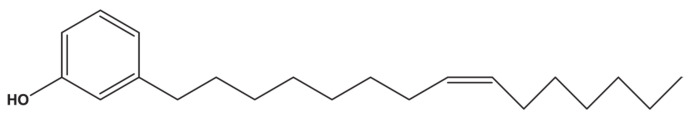
Structure of cardanol.

Cardanol is obtained from cashew nut shell liquid (CNSL), a byproduct of the cashew nut processing industry. CNSL is a mixture of anacardic acid (71.7%), cardanol (4.7%), traces of cardol (18.7%), 2- methylcardol (2.7%), and unidentified polymeric material (2.2%). Cardanol is extracted from CNSL by pyrolysis, heat and supercritical carbon dioxide extraction, or by solvent extraction. Extracted CNSL contains a higher percentage of cardanol. The extraction techniques of CSNL and its characterizations have been explained in detail in a recent review [101]. The structure of cardanol is shown in Figure 4.

Due to the presence of the phenolic groups on cardanol, it has been used as a natural substitute for phenol in the production of PF resin. Both novolac and resol resins have been synthesized with partial substitution of phenol with cardanol. The percentage of substitution influences the mechanical and thermal properties of the resulting resin. Cardanol substitution has been found to reduce thermal stability and tensile strength in some PF resins [102,103]. Parameswaran et al. developed resol resin by substituting phenol with cardanol in various molar ratios (ranging from 1:0 to 0.9:0.1) with formaldehyde, and the tensile and flexural strengths of the resin were found to decrease with increasing cardanol substitution [104]. The lower melt viscosity of cardanol facilitates processing and the long and unsaturated side chain affects mechanical and thermal properties. Liang et al. oxidized the unsaturated side chain of cardanol to hydroxyl, to improve the hydrophilicity of the resulting resin, with the aim of improving the foam production. Polyhydroxylated cardanol (PHC) was used to replace 20% of phenol in the resol resin production and the synthesized resin was successfully used to prepare PF foams with excellent mechanical properties. PHC substitution has been found to increase the compressive strength and flexural strength by 57% and 56% respectively [105]. To improve the thermal stability and sustainability of phenolic resin, cardanol-substituted phenolic resin was prepared by copolymerizing salicyl alcohol, cardanol, and boric acid with the phenol-formaldehyde-cardanol mole ratio of 1:1.2:0.05. The boron content increases the thermal stability of the resin [106]. Natarajan et al. developed another method to improve the thermal stability of cardanol substituted resin. The novolac type resin was synthesized from cardanol, and formaldehyde with the mole ratio of 1:0.7 using oxalic acid as the catalyst, and to increase the thermal stability, the resin was epoxidized using epichlorohydrin [107]. Another research group studied the thermal degradation kinetics of the epoxidized cardanol resin [108]. Jadhav et al. adopted a rare synthetic route to prepare cardanol based novolac resin. An energy efficient and time-saving ultrasound-facilitated synthesis method was utilized for preparing novolac resin, resulting in a reduction of reaction time from 5 h to 30 min compared to the conventional synthesis [109]. To improve the properties of the cardanol-formaldehyde resin, it was modified by substituting formaldehyde with furfural. The mechanical, chemical, and curing characteristics of cardanol–furfural resin were improved after furfural substitution [110].

Cardanol-substituted resin and their performance characteristics are summarized in Table 3.

## 4. Formaldehyde Substitutes for PF Resin

Formaldehyde has been extensively used as a key raw material in a wide variety of industries and manufacturing fields. The main consumption areas are the wood industry, flooring materials, lubricants, cosmetics, medicines, insulation materials, disinfectants and cleaning products, preservatives, paper, and photo processing. It is widely used in indoor and outdoor applications, and people in all areas of life are exposed to it in one way or other. However, the health risks of formaldehyde are of major concern due to the widespread use of formaldehyde in various sectors of life, a number of scientific studies have been conducted to understand the health implications [112,113,114].

Formaldehyde is also harmful to the environment as it affects plant life and wildlife. Due to the environmental and occupational concerns of formaldehyde, a number of regulations and advisories are established to limit the exposure level. The occupational exposure limit of formaldehyde varies between countries as shown in Table 4.

Even though formaldehyde is an important resource in many vital industries, its adverse impact on human health have driven industries and researchers to find safer and more environmentally benign alternatives. Bio-based alternatives would the most significant replacement option for formaldehyde. Hydroxymethylfurfural, furfural, furfuryl alcohol, glyoxal and vanillin are some of the bio-based molecules that have been investigated for replacing formaldehyde in PF resin synthesis.

### 4.1. Hydroxymethylfurfural-Substituted Resin

Hydroxymethylfurfural (HMF) is considered by many as the best alternative for formaldehyde and holds a high potential since it can serve as a source for many other chemicals and liquid fuels. It is an organic compound consisting of a furan ring with an aldehyde and alcohol functional group. HMF is highly reactive and soluble in aqueous media due to the presence of aldehyde and alcohol functional groups. HMF acts as a precursor in the synthesis of liquid alkanes [120], cosmetics, polymers, pharmaceuticals, and agrochemicals industries and shows antioxidant activity, protection activity against hypotoxic injury, anti-allergen activity, and act as an anti-sickling agent [121,122,123]. The industrial potential of HMF is significant as it can be used for the production of dimethylfuran (DMF) and ethoxymethylfurfural (EMF), two biofuel candidates for automatic vehicles [124,125]. Several other chemicals including levulinic acid (LA) [126], succinic acid [127], and 2,5-furandicarboxylic acid [128] can also be synthesized from HMF by various chemical reactions including oxidation, hydrogenation, etherification, and rehydrogenation.

**Figure 5:**
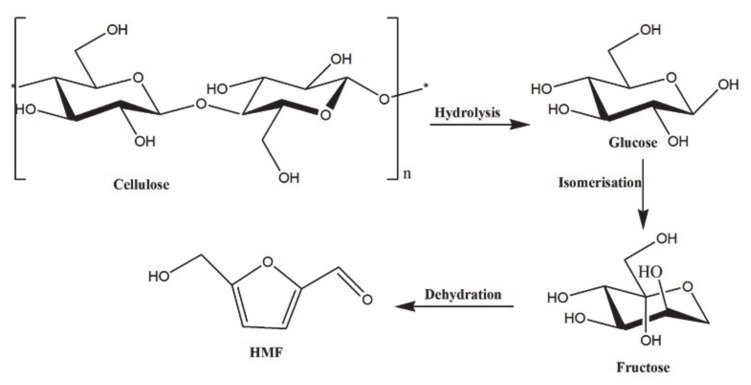
Synthesis of hydroxymethylfurfural (HMF) from cellulose.

HMF can be synthesized from lignocellulose and cellulose with the help of organic solvents and ionic liquids [129]. It is an aromatic aldehyde present in dried fruits, honey, coffee, and flavoring agents. The percentage of HMF in these bioresources varies, and catalytic dehydration has been widely used to extract HMF from them [130]. The main bioresources for the production of HMF are carbohydrate materials such as simple sugars and their derivatives, and carbohydrate polymers such as lignocellulose, cellulose, lignin, inulin, and starch. Mainly non-food plant resources are used in the production of HMF. Inulin is extensively used to produce HMF by a variety of methods including an ionic liquid-based process or the Fenton reaction process, for example. Catalysts capable of converting biomass into HMF have been developed in recent years [131,132]. The first step in the conversion of lignocellulose into HMF is the depolymerization or hydrolysis, which helps to break the bonds between the polymers and thereby helps for the catalyst reaction [133]. Detailed mechanism and reagents/catalysts used for converting lignocellulose/cellulose into HMF has been explained in recent reviews [134,135]. The schematic diagram for the synthesis of hydroxymethylfurfural from cellulose is shown in Figure 5. 

Zhang and coworkers developed a number of PF resins by substituting formaldehyde with HMF. The group converted glucose into HMF via an in-situ process using CrCl_2_/CrCl_3_ and tetraethylammonium chloride (TEAC) catalysts. A number of catalysts based on Cr and Zr have been used for the conversion of glucose into HMF. HMF was generated in situ from glucose in the presence of CrCl_2_/CrCl_3_/TEAC (tetraethylammonium chloride) catalysts and reacted with phenol to prepare the resin. The resin cured using the common curing agent hexamethylenetetramine (HMTA) and used to prepare fiber glass reinforced composite. The thermal, mechanical, and curing characteristics of the composite were evaluated as a function of the HMTA concentration. This study demonstrated that HMF modified PF resins can be used for making green composites with zero formaldehyde emission upon heating [136]. In order to prepare a resin completely from sustainable resources, they replaced phenol with a bio phenol (hydrolysis lignin) and the corresponding resin was prepared by reacting with HMF and curing with HMTA. The resin exhibited good thermal stability up to 315 °C [137]. Even though HMTA has been extensively used for curing novalac-type phenolic resin, it also has environmental concerns as it is susceptible to decomposition at room temperature, releasing toxic chemicals [138]. Therefore, the researchers replaced HMTA with an environment-friendly curing agent, organosolv lignin (OL) or Kraft lignin (KL). The thermal stability, flexural strength, and tensile strength of the resin were found to be comparable to the HMTA cured phenol-hydroxymethylfurfural resin. The OL/KL cured PHMF prepared resin finds its application in fiberglass reinforced composite materials. This study demonstrated the possibility of utilizing OL/KL as curing agents for such resins [139].

Another curing agent, Bisphenol A type epoxy resin, i.e., bisphenol A diglycidyl ether (DGEBA), has also been used instead of HMTA to crosslink the phenol–hydroxymethylfurfural (PHMF) resin. A bio-based novolac-type resin—phenol–hydroxymethylfurfural (PHMF) resin—was prepared by reacting phenol with HMF, in-situ derived from glucose, at 120 °C by acid catalysis. Bisphenol A type epoxy resin, i.e., bisphenol A diglycidyl ether (DGEBA), was used as a formaldehyde-free curing agent by substituting conventional formaldehyde-based hexamethylene tetraamine (HMTA) to crosslink the PHMF resin. The thermal and mechanical properties of the cured resin make it useful for fiber reinforced plastics/composites [140].

### 4.2. Furfural-Based Resin

**Figure 6:**
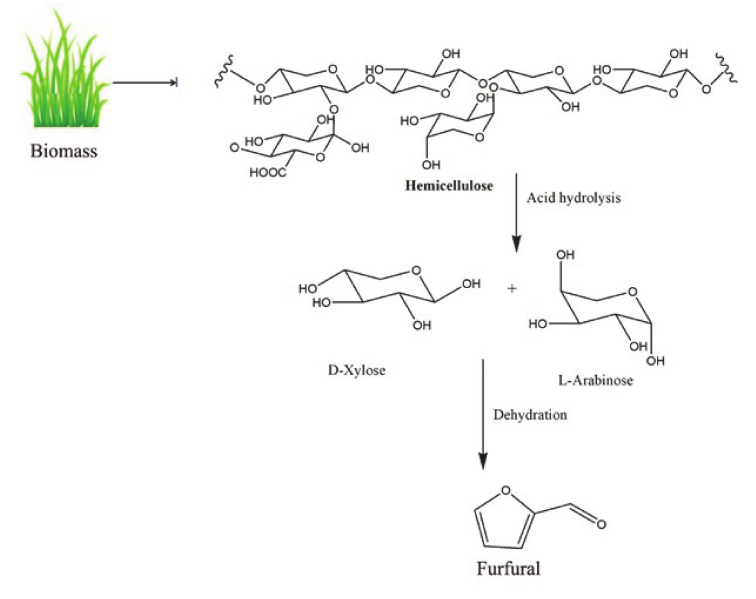
Schematic representation of furfural synthesis from biomass.

Furfural is an organic compound consisting of a furan ring with an aldehyde functional group on the 2 position of the ring. Furfural has industrial value in resin production, as a lubricant, and in the synthesis of several other organic compounds such as tetrahydrofuran, furfuryl alcohol (FFA), tetrahydrofurfuryl alcohol (THFA), methyltetrahydrofuran (MTHF), furfurylamine, furoic acid, and methylfuran [141]. Furfural is produced from non-food residues of wood waste. Industrial production of furfural started in 1921 by Quaker Oats Company. The process involves conversion of lignocellulosic residues containing pentosans into pentoses by acid hydrolysis followed by steam stripping (dehydration) into furfural. This method was not economically feasible due to low yield and high production cost, which eventually led to the closure of the manufacturing plants in the 1990s. Companies in China, Australia, and Netherlands produced furfural from lignocellulose biomass by modified procedures with better yield. The industrial and lab scale production of furfural has been explained in a recent review [13]. Ionic liquids have also been used to produce furfural from lignocellulose materials. Peleteiro et al. recently reviewed the ionic liquids used for the conversion of lignocellulose into furfural [142]. The schematic representation of furfural synthesis from biomass is shown in Figure 6.

Phenolic resins have also been developed from other aldehydes. Carbohydrates have been modified to furfurals to prepare resin with phenol. Pizzi and coworkers developed phenol-furfural resin and their flow properties and electrical properties were studied [143,144]. The resin has been used as wood adhesives [145], and also for cold-setting binders for foundry core sand [146]. Oliveira et al. prepared the phenol-furfural resin by a previously reported procedure, and their detailed structural characterization was performed using NMR and MALDI-TOF spectroscopy, revealing that the structure contains more linear oligomers than cyclic ones. Composites were made from the phenol-furfural resin and sisal fibers. Thermal studies such as thermogravimetric analysis (TGA) and differential scanning calorimetry (DSC), as well as electron microscopy, demonstrated the excellent adhesion between the resin and sisal fibers [147].

Cheng et al. reported the synthesis of phenol substituted bio-oil–phenol–formaldehyde (BPF) adhesives using furfural as the crosslinking agent. With an increase in furfural loading, the methylene bridges between phenol and furfural increased and, therefore, the bonding strength of the highly substituted resin improved. The wet tensile strength of the BPF resin increased to 2.84 MPa compared to 1.54 MPa for PF resin [148]. Riya et al. developed phenol furfural resin from moringa oleifera gum and biophenol and studied its application in styrene butadiene rubber. Resin was synthesized by varying reaction conditions such as temperature, time, and catalyst type. The prepared resin was characterized by FT-IR, NMR, TGA, and DSC. The resin improved the tack and the rheological properties of the rubber [149]. Formaldehyde was completely replaced by furfural and glucose in a recently reported research work. Phenol–furfural–glucose (PFuG) resol type resins with different molar ratios of glucose to furfural were developed for use as wood adhesive and, the key properties such as bonding strength, free phenol, pH, and solid content of the resin were analyzed. These were found to meet the standard requirement for wood adhesives as per the GB/T 14732-2006 standards [28].

Ahuja et al. studied the kinetics of phenol-furfural novalac resins in the presence of potassium carbonate catalyst with a wide range of furfural to phenol (F/P) mole ratio. They postulated a reaction scheme applicable to alkali catalyzed phenol-furfural novalac resins. The study focused on the effects of the mole ratio of the reactants and the functionality of the phenol on the rate constant. The specific rate constant was found to be independent of the initial mole ratio of the reactants [150]. Dongre et al. replaced phenol and formaldehyde used in PF resins by lignin and furfural respectively. Lignin recovered from acid hydrolysis of hot water extract was cross-linked with furfural. The blend prepared at pH = 1 with 16% furfural exhibits good tensile properties the PF tensile strength [151].

### 4.3. Glyoxal-Based Resin

Glyoxal is another aldehyde used to replace formaldehyde in phenolic resin synthesis. It is a nontoxic aldehyde, with two aldehyde groups in its structure, which make it highly reactive. The non-volatility, low cost, and easy biodegradability make it a suitable candidate for formaldehyde substitution in phenolic adhesive manufacturing. Glyoxal is present in dietary products such as wine, beer, tea, coffee, yogurt, bread, rice, soybean paste, soy sauce, and oil.

**Figure 7:**
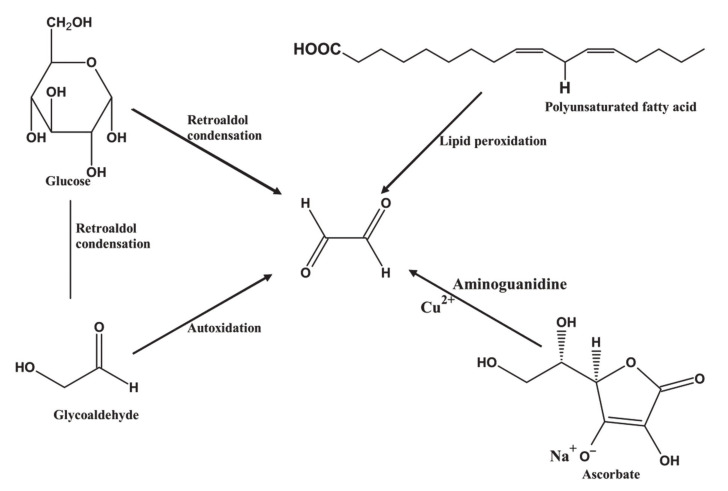
Synthesis of glyoxal from carbohydrate resources.

It can be prepared from glucose directly via retroaldol condensation, and indirectly from a glycoaldehyde intermediate by autoxidation [152]. Glyoxal can also be prepared from galactose, mannose, fructose, ribose, arabinose, ribulose, glyceraldehyde, acetone, adenosine, mannitol, and glycerol [153]. Synthetic routes used for the preparation of glyoxal are shown in Figure 7.

Glyoxal is obtained by the oxidation of lipids or as a byproduct of biological processes [154], and has been used to replace formaldehyde in many phenolic resins [28]. A number of phenolic resins were prepared by replacing phenol with environment friendly lignin. Both raw and modified lignin were used for replacing phenol in the production of phenolic resin and a number of modification methods were reported. Younesi-Kordkheili et al. synthesized a new adhesive by reacting phenol, lignin, and glyoxal and the physical and mechanical properties of the particleboards prepared with the resin (PLG resin) were evaluated. The PLG resins with higher percentages of lignin had slower gel time and lower viscosity compared to the control PF resin. PLG resin with 30 wt % lignin substitution was found to need a higher curing temperature. When lignin content was increased from 20 to 40 wt %, plywood panels bonded with PLG resins shows weaker mechanical properties and lower dimensional stability, bending, and internal bond strengths [155]. Younesi-Kordkheili used ionic liquids for the modification of lignin and used it to prepare lignin-phenol-glyoxal resin. The properties of the resins indicated that lignin modification with ionic liquid accelerates the gelation time and increases viscosity, density, and solid content and decreases the curing temperature [156]. Hussin et al. isolated lignin from Kenaf via soda and Kraft pulping and prepared SLPG (soda lignin-phenol-glyoxal) and KLPG (Kraft lignin-phenol-glyoxal) adhesives respectively and their properties were compared to phenol–formaldehyde (PF) resin. Soda lignin provides more cross-linking with glyoxal than Kraft lignin due to the higher number of phenolic –OH groups in its structure. Hence, the soda lignin-based resins have the highest percentage solid content, higher viscosity, and shortest gel time, higher tensile strength (72.08 MPa), elongation break, and internal bonding (53.83 N mm^−2^); thus, they could be a suitable replacement for PF resins in the wood adhesive industry [157]. Aziz et al. extracted lignin from coconut husk via Kraft and soda pulping. Kraft lignin (KL) and soda lignin (SL) were characterized by different techniques. Both lignins were used to produce resins with glyoxal by partially replacing the phenol. The physico-chemical and physico-mechanical properties of the KLPG and SLPG resins were analyzed and it was revealed that the 30% SLPG resin shows better properties than KPLG resin, due to the phenolic and G-type unit in the lignin structure. Addition of layered double hydroxides (LDH) as reinforced filler into SLPG resin further improved the properties. For example, LDH addition from 3 to 15 wt % increased the MOE of LDH-SLPG resins rapidly from 680 to 852 MPa. This study identified that lignin isolated from a coconut husk could be a suitable substitute for phenol [158]. Rao et al. prepared lignin-resorcinol-glyoxal resin and used it for the preparation of lightweight, hydrophobic, and recyclable carbon foam using PU foam as a substrate [159]. 

El Mansouri et al. completely replaced formaldehyde with glyoxal in a lignin-based adhesive. Wood panels manufactured with this adhesive demonstrated high internal bond strength [160]. Phenol–formaldehyde resin was prepared by replacing formaldehyde with glyoxal and reinforcing with sisal fibers (30%, w/w). The resin was cured at two different temperatures—cycle 1 limited the curing temperature to 150 °C, whereas cycle 2 limited the temperature to 180 °C. Composites molded with cure cycle 1 were found to have high storage modulus and the composite molded with cure cycle 2 had a high cross-linking density. [161]. Ammar et al. substituted formaldehyde with glyoxal and the stability of Green jelutong (*Dyera costulata*) wood was enhanced by using glyoxyated lignin formaldehyde resin as a bulking agent [162]. Formaldehyde substitutes used in the synthesis of phenolic resin are summarized in Table 5.

## 5. Technical and Economic Challenges of Bio-Based Phenolic Resin

Phenolic resins made from bioresources have been developed and studied extensively in recent decades. However, their industrial production and applications are still at a relatively early stage of development. Even though sustainable resources are necessary to replace the depleting natural petroleum resources, technical and economic challenges associated with the bioresources are the factors that must initially be overcome for their industrial applications [164]. Biobased phenol substitutes such as lignin, cardanol, and tannin contains large numbers of phenolic groups needed for the reaction with formaldehyde, but most of them require structural modifications for high reactivity for resin synthesis [165,166]. The properties of resin synthesized from bioresources are inferior to petroleum-based resin in many cases. Most of the bioresources are the byproducts of biorefineries and special methods are required for their separation and purification. For example, lignin, the most abundant byproduct of biorefineries, requires significant purification and even structural modification prior to its use in resin production [167]. In addition to that, the structure of the lignin varies considerably depending on its origin in terms of number of functional groups, molecular weight, and the degree of cross linking. All of the steps required to increase the reactivity of lignin escalate the production cost.

## 6. Conclusions

Phenolic resin continues to garner applications in all sectors of the building material industry, including the construction, engineering, and technology sectors, even a hundred years after its first synthesis. The high-end mechanical and thermal properties make the resin an inevitable choice for many industrial applications. Research is continuing to enhance the properties and widen the end-use of phenolic resin and derived products. However, petroleum product depletion, continuous fluctuations in fuel price, and increasing focus on health issues associated with the materials used to produce PF resin have driven the search for safer and more sustainable resources for resin synthesis. In this context, phenolic resins have been produced from bioresources and used as adhesives in the wood industry. Wood panels produced from modified PF resin show similar or superior properties compared to those developed from conventional phenolic resin, while also representing an environmentally friendly alternative to traditional PF resin. This review article gives an insight into the bio-based alternatives for phenol and formaldehyde used in the production of resin. These natural chemicals can significantly reduce the harmful impacts on the environment and human health. The literature provided in this article gives the reader an overview of the current status of bio-based phenolic resin and provides valuable information for further investigation in this area. Although these bio-based resins are considered environmentally friendly, bio-based resources substitution sometimes decreases the mechanical properties of the resin compared to the conventional phenolic resin. Furthermore, there are significant challenges in the development of industrial scale processes for these modified resins, due to variability in chemical composition of the bioresources used and the need for multiple purification and modification steps. Therefore, future research on improving the sustainability of PF resins will be focused on enhancing their properties, especially mechanical aspects, improving the preparation of their raw materials from bioresources, and broadening their application in a variety of industries.

## Figures and Tables

**Table 1:** Examples of lignin substituted PF resins.

Substitute	PF Resin Type	% Substitution	Effect on Performance	Ref.
Kraft Lignin	Resol	90	Increase in adhesive strength and decrease in gel time	[67]
Organosolv wheat straw lignin	Resol	50–70	Resin achieved the specification of PF resin in terms of pH, viscosity, and solid content	[68]
Organosolv pine lignin	Resol	25–75	Substitution up to 50% decreases the curing temperature of the resin	[69]
Corn stover lignin	Resol	100	Mechanical properties similar to PF resin	[70]
Methylolated softwood ammonium lignosulfonate	Resol	30	High thermal stability and modified rheology	[74]
Enzymatic hydrolysis lignin	Novolac	55	Low free phenol and longer gelation time	[76]
Kraft pine lignin, Soda/anthraquinone flax lignin, and Sulfonated kraft lignin	Novolac	25–45	Increase in flexural modulus	[77]
Kraft lignin	Novolac	25–45	Low gelation time	[78]

**Table 2:** Examples of tannin substituted phenolic resin.

Substitute	PF Resin Type	% Substitution	Effect on Performance	Ref.
Valonea Tannin	Resol	30	Short curing time, good bonding strength and low formaldehyde emission	[81]
Tannin	Resol	20	Low formaldehyde emission and could meet the requirement of GB/T17657-2013	[90]
Condensed tannin	Resol	10–30	Lower PH, higher viscosity and shorter gel time	[91]
Chestnut tannin	Resol	30	Increase in compressive strength	[92]
Larch Tannin	Resol	30	Friability and thermal conductivity increased with increase in tannin content	[99]
Chestnut tannin	Novolac	4–40	Short gelation time	[100]

**Table 3:** Examples of cardanol substituted phenolic resin.

Substitute	PF Resin Type	% Substitution	Effect on Performance	Ref.
Cashew nut shell liquid	Resol	10–90	Improvement in impact strength and electrical properties	[103]
Polyhydroxylated cardanol	Resol	20	Increased in compressive strength and flexural strength.	[105]
Cardanol	Resol	100	Thermal stability increased	[108]
Cardanol	Novolac	100	Reduction in reaction time	[109]
Cashew nut shell liquid	Novolac	100	Increase in thermal stability	[111]

**Table 4:** The permissible limit of formaldehyde in various countries.

Country	OEL (ppm)					Reference	
	TWA		STEL		TLV		
Australia	1		2			[115]	
China					0.4	[116]	
Germany	0.3					[117]	
Japan	0.1					[118]	
South Africa	1		2			[116]	
United Kingdom	2		2			[116]	
United States	0.75				2	[119]	

TWA—Time-weighted average. STEL—Short term exposure limit. PEL—Permissible exposure limit. TLV—Threshold limit value.

**Table 5:** Formaldehyde substitutes used in the synthesis of phenolic resin.

Substitute	PF Resin Type	% Substitution	Performance	Ref.
Hydroxymethylfurfural	Novolac	100	Increase in tensile strength	[163]
Hydroxymethylfurfural	Novolac	100	High thermal stability and increased curing time	[137]
Hydroxymethylfurfural	Novolac	100	High tensile strength and glass transition temperature	[140]
Furfural	Resol	100	Increased in tack and the rheological properties of the rubber	[149]
Furfural	Resol	100	High bonding strength, thermal stability, and low free phenol content	[75]
Furfural	Resol	5 to 15	Wet tensile strength increased	[148]
Glyoxal	Resol	100	Good internal bond strength	[160]
Glyoxal	Resol	100	Curing time can be varied	[161]
Glyoxal	Resol	100	Greater mechanical strength and dimensional stability	[156]
